# Construction of a Signature Model to Predict the Radioactive Iodine Response of Papillary Thyroid Cancer

**DOI:** 10.3389/fendo.2022.865909

**Published:** 2022-05-11

**Authors:** Lina Liu, Yuhong Shi, Qian Lai, Yuan Huang, Xue Jiang, Qian Liu, Ying Huang, Yuxiao Xia, Dongkun Xu, Zhiqiang Jiang, Wenling Tu

**Affiliations:** ^1^ Department of Nuclear Medicine, the Second Affiliated Hospital of Chengdu Medical College, China National Nuclear Corporation 416 Hospital, Chengdu, China; ^2^ School of Bioscience and Technology, Chengdu Medical College, Chengdu, China; ^3^ Department of General Surgery, the Second Affiliated Hospital of Chengdu Medical College, China National Nuclear Corporation 416 Hospital, Chengdu, China

**Keywords:** papillary thyroid cancer, radioactive iodine refractory, RNA signature, prognosis, model

## Abstract

Papillary thyroid cancer (PTC) accounts for about 90% of thyroid cancer. There are approximately 20%–30% of PTC patients showing disease persistence/recurrence and resistance to radioactive iodine (RAI) treatment. For these PTC patients with RAI refractoriness, the prognosis is poor. In this study, we aimed to establish a comprehensive prognostic model covering multiple signatures to increase the predictive accuracy for progression-free survival (PFS) of PTC patients with RAI treatment. The expression profiles of mRNAs and miRNAs as well as the clinical information of PTC patients were extracted from TCGA and GEO databases. A series of bioinformatics methods were successfully applied to filtrate a two-RNA model (IPCEF1 and hsa-mir-486-5p) associated with the prognosis of RAI-therapy. Finally, the RNA-based risk score was calculated based on the Cox coefficient of the individual RNA, which achieved good performances by the time-dependent receiver operating characteristic (tROC) curve and PFS analyses. Furthermore, the predictive power of the nomogram, integrated with the risk score and clinical parameters (age at diagnosis and tumor stage), was assessed by tROC curves. Collectively, our study demonstrated high precision in predicting the RAI response of PTC patients.

## Introduction

Among various types of endocrinal malignancy, thyroid cancer or carcinoma (TC) accounts for the highest incidence rate, in both women and men, and its yearly occurrence has been alarmingly increasing worldwide (1, 2). A global statistics on the TC-related deaths shows 44,000 deaths and 586,000 new cases reported in the 2020 database (3). Papillary thyroid cancer (PTC) is histologically the most well-differentiated TC including about 90% of all TCs (2). PTC generally has an excellent prognosis with a 10-year survival rate between 80% and 95% when treated by conventional thyroidectomy and adjuvant radioactive iodine (RAI) therapy to ablate the residual thyroid tissue and thus the chances of metastasis (4, 5). RAI therapy has recently been evolved exhibiting much higher prognostic outcomes relative to conventional treatments in PTC patients (5–7). However, approximately 20%–30% of PTC patients show either resistance to RAI therapy and/or recurring PTC events (8, 9). For these subsets of patients, despite multiple treatment modalities, including radio- and chemotherapy, thyroidectomy, and targeted therapy, the 10-year survival rate could not reach 10% (4, 10). Hence, early identification of PTC and genetic screening of patients are crucial to distinguishing the ones refractory to RAI therapy.

The sodium-iodide symporter (NIS) is a trans-membrane glycoprotein having 13 transmembrane domains that mediates the active uptake of each circulating iodide ion in exchange of 2 intracellular sodium ions by the thyroid gland (11). It has been found that RAI resistance is related to the abnormal depletion or decreased expression of NIS (12, 13). Multiple mechanisms have been identified in connection to altered NIS expression in PTC patients, such as thyroid gland-specific transcription termination factor-1 (TTF1) and paired box gene-8 (PAX8)-mediated induction of thyroid-stimulating hormone (TSH), which in turn modulates NIS transcription (14). Furthermore, cancer-causing mutations in any of Ret Proto-oncogene (*RET*), neurotrophic tyrosine receptor kinase (*NTRK*), RAS, B-Raf proto-oncogene (*BRAF*), or telomerase reverse transcriptase (*TERT*) gene are linked to loss of thyroid-differentiating genes, including NIS (15–17). Among them, BRAF^V600E^ mutation is the most frequently observed genetic alteration. Several studies have suggested a possible linkage between BRAF^V600E^ mutation and RAI refractiveness in PTC recurrence, metastasis, and poor prognosis (15, 16, 18). However, there have been contradictory findings showing no apparent cross talk between mutant BRAF and PTC pathology (19–21), suggesting that BRAF mutation may not be an independent prognostic factor in predicting an RAI non-responsive PTC population. Thus, it is very important to explore new and effective prognostic factors to predict the RAI response in PTC patients.

Moreover, dysregulated RNAs have been implicated in contributing to the occurrence, metastasis, and prognosis of PTC (9, 22–24). For example, a higher expression of interleukin 37 (IL37) and a lower expression of HIG1 hypoxia-inducible domain family member 1B (HIGD1B), polypeptide N-acetylgalactosaminyltransferase 9 (GALNT9), and serum deprivation-response protein (SDPR) in PTC tissues predict worse outcomes (24, 25). In addition, higher expressions of differentially regulated miRNAs, including miR-221, miR-146, miR-193, miR-182, miR-486, and miR-564, are correlated with increased risk of metastasis and poor prognosis in PTC (9, 22, 23, 26). Considering the genetic diversity in cancer patients, it has been postulated to develop an array of prognostic indicators, instead of relying on a single marker, for better prediction of the overall survival (OS) rate in a patient-specific manner; e.g., Ma et al. have reported a signature panel of six genes (*AZGP1*, *IGF2BP2*, *MEX3A*, *NUDT16*, *NUP153*, and *USB1*) to predict OS in PTC (27). Furthermore, a recent study has demonstrated that an eight-gene (*ULBP2*, *S100A5*, *LTF*, *PLXNA4*, *FAM3B*, *GIPR*, *RORB*, and *TGFBR3*) prognosis model is associated with progression-free survival (PFS) of PTC patients (28). However, no model exists to predict the prognosis in PTC patients undertaking RAI therapy.

**Figure 1:**
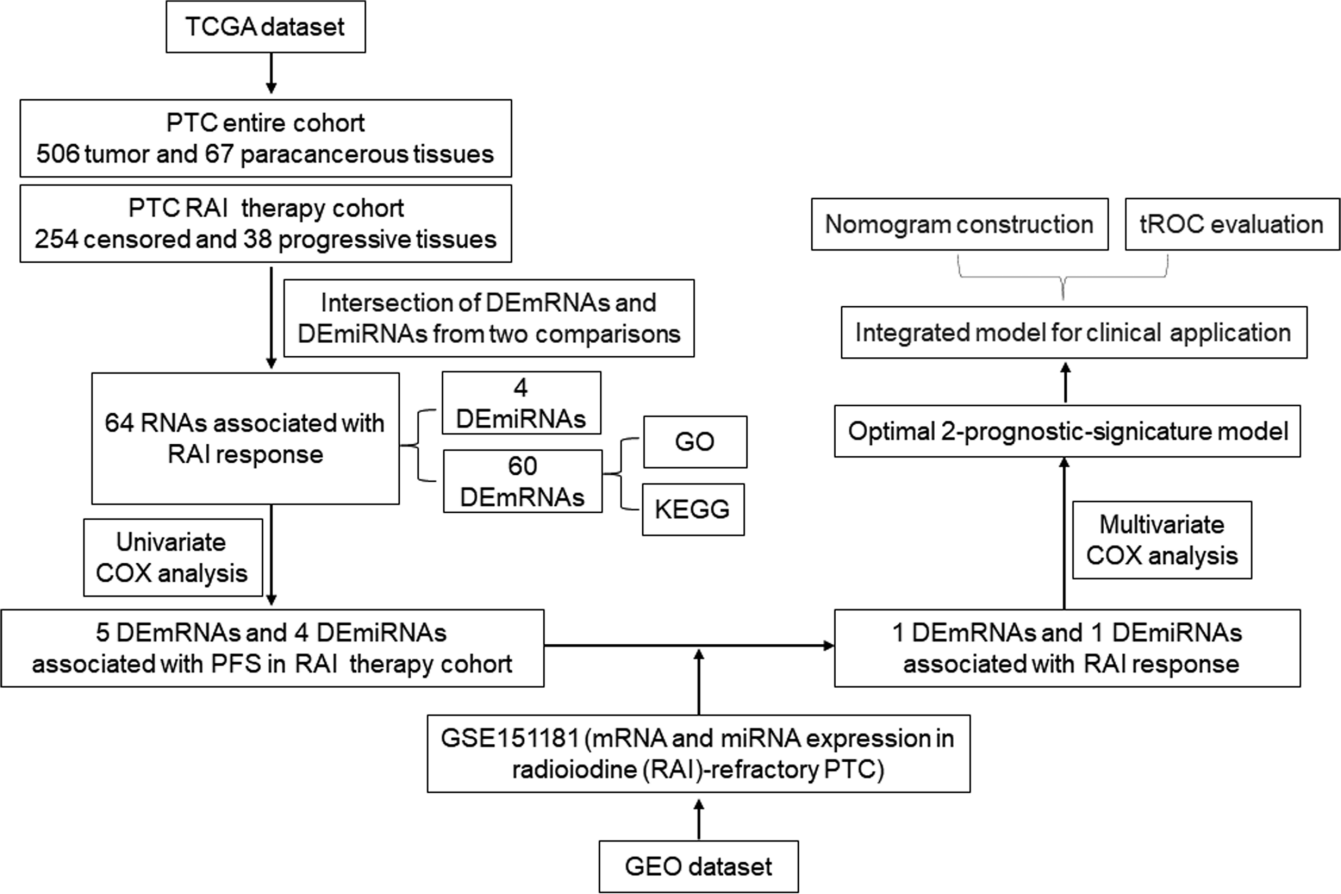
The workflow of the bioinformatic analysis.

Here, we demonstrate the development of a prognosis prediction model based on the RNA biomarkers and the most relevant clinical parameters to efficiently predict the PFS of PTC patients with RAI therapy. We chose PFS instead of OS because PFS refers to two key factors (recurrence and metastasis) resulting in poor prognosis of PTC patients, which was more valuable to reflect the disease state of PTC. PFS was defined as the time of diagnosis to first tumor progression or death of any cause. According to the clinical information, the tumor progression was defined by disease-free status (PFS = 0: censored; PFS = 1: progression) or overall survival status (OS = 0: living; OS = 1: deceased). For this, we selected differentially expressed (DE) miRNA and mRNA profiles in PTC tissues from The Cancer Genome Atlas (TCGA) and Gene Expression Omnibus (GEO) databases and ran multidimensional and multi-perspective analyses with massive bioinformatics methods, which eventually identified a two-RNA group as the significant prognostic indicator of PFS of PTC patients with RAI therapy. First, the multivariate Cox coefficient multiplied by a relative fold change in RNA expression was used to calculate the risk score, which was then combined with clinical parameters to construct a nomogram. Finally, the time-dependent receiver operating characteristic curve (tROC) analysis was used to assess the nomogram. The workflow of model development is shown in **Figure 1**.

## Materials and Methods

### Characteristics of the Datasets

Patients’ clinical information and molecular profiles including miRNA and mRNA expressions were downloaded from TCGA database (https://cancergenome.nih.gov/). The entire cohort included 506 PTC tumor and 67 para-cancerous samples. According to 2015 American Thyroid Association (ATA) guidelines for DTC (4), the disease-free status comprises all of the following: (a) no clinical evidence of tumor; (b) no imaging evidence of tumor by RAI imaging (no uptake outside the thyroid bed on the initial posttreatment WBS if performed, or if uptake outside the thyroid bed had been present, no imaging evidence of tumor on a recent diagnostic or posttherapy WBS) and/or neck US; and (c) low serum Tg levels during TSH suppression (Tg <0.2 ng/ml) or after stimulation (Tg <1 ng/ml) in the absence of interfering antibodies. Otherwise, it is defined as tumor progression. RAI-refractory is defined as any of the following: (i) the malignant/metastatic tissue does not ever concentrate RAI (no uptake outside the thyroid bed at the first therapeutic WBS); (ii) the tumor tissue loses the ability to concentrate RAI after previous evidence of RAI-avid disease (in the absence of stable iodine contamination); (iii) RAI is concentrated in some lesions but not in others; and (iv) metastatic disease progresses despite significant concentrations of RAI. Otherwise, it is defined as RAI-sensitive. In this study, it is impossible to obtain the standard information of disease-free status and RAI-refractory as defined by ATA guidelines from TCGA database. To solve this problem, 292 PTC patients with “RAI therapy=Yes” were screened out. Among them, patients satisfying the “PFS = 0: censored” condition comprised the RAI-sensitive group (n = 254), and the remaining 38 subjects meeting “PFS = 1: progression” criteria were included in the RAI-refractory group (n = 38).

The only relevant microarray dataset GSE151181 related to RAI-refractory PTC and corresponding clinical information were obtained by searching all available data related to RAI-refractory PTC in the GEO database (http://www.ncbi.nlm.nih.gov/geo/). GSE151181 was performed on GPL23159 [Clariom_S_Human] Affymetrix Clariom S Assay, Human (Including Pico Assay), and GPL21575 Agilent-070156 Human_miRNA_V21.0_Microarray 046064 (Feature Number version). Based on the inclusion criteria for RAI-refractory PTC patients, the following samples were: before RAI, 11 non-neoplastic thyroid tissues, 13 primary tumors, and 5 synchronous lymph node metastases, and 17 lymph node metastases post RAI.

### Screening of DEmRNAs and DEmiRNAs Associated With RAI Response

The “edgeR” R package was used to screen DE mRNAs (DEmRNAs) and miRNAs (DEmiRNAs) in 506 PTC tumor and 67 para-cancerous samples. The cutoff thresholds for selection of DEmRNAs were P < 0.05 and |log2 fold change (FC)| ≥1, while those for DEmiRNAs were |log2 FC| ≥0.8 and P < 0.05.

The PTC tumor samples with RAI therapy were further divided into RAI-refractory group (n = 38) and RAI-sensitive group (n = 254), according to the RAI response. The overlap between the two comparisons was considered as DEmRNAs and DEmiRNAs associated with the RAI response.

### Functional Enrichment Analysis

The Kyoto Encyclopedia of Genes and Genomes (KEGG) pathway and Gene Ontology (GO) analyses were enriched by the Metascape database (http://metascape.org/). The GO analysis mainly described three terms: molecular function (MF), biological process (BP), and cellular component (CC). GO terms or KEGG pathways with a P-value < 0.05, a minimum count of 3, and an enrichment factor >1.5 were considered statistically significant.

### Screening of DEmRNAs and DEmiRNAs to Predict Prognosis in RAI Therapy

After excluding patients with incomplete clinical information, a total of 494 PTC patients were listed with detailed clinical information, of which 289 patients were treated with RAI and 178 patients did not receive RAI therapy, and 27 patients had incomplete records. Univariate Cox regression analysis was applied to evaluate the overlapping DEmRNA and DEmiRNA factors associated with PFS in the PTC with RAI therapy cohort (n = 289). The RAI-associated DEmRNAs and DEmiRNAs significantly associated with PFS were considered as the first candidate RNA sets. Subsequently, GSE151181 was used to validate the expression levels of these RNAs among normal group (11 samples of non-neoplastic thyroids before RAI), before RAI group (13 samples of primary tumors and 5 samples of synchronous lymph node metastases), and after RAI group (17 samples of lymph node metastases). After validation, DEmRNAs with P < 0.05 and |log2 FC| ≥1, and DEmiRNAs with P < 0.05 and |log2 FC|≥0.8, were included in the second candidate RNA sets. To further verify the prognostic power of the second candidate RNA sets, Kaplan–Meier survival curve analysis and log-rank (LR) test were used to measure the PFS differences between the high-expression and low-expression groups in the PTC RAI therapy cohort, without RAI therapy cohort, and the entire PTC cohort, respectively. The validated DEmRNAs and DEmiRNAs were screened out as the final RNA sets.

### Construction and Evaluation of a Prognostic Model for PTC Patients With RAI

Individual patients’ PFS risk score was determined by multiplying the multivariate Cox regression coefficient with the relative expression level of the signature RNAs. Hence, the risk score was calculated as follows: Risk score = (signature1 coefficient × signature1 expression) + (signature2 coefficient × signature2 expression) + ⋯ + (signature N coefficient × signature N expression). The linear combination format for risk score calculation for individual target RNA as follows:


$$Risk\;score\;=\sum_{i=1}^{3}\beta_{i}*Ex{\text{p}}_{i}$$

Exp indicates the candidate RNA expression level, and *β* is the corresponding regression coefficient.

All 289 patients with RAI therapy were distributed into high-risk and low-risk groups based on their median risk score values. Then, Kaplan–Meier survival curve analysis with the LR test was performed to compare the PFS differences between these two groups. A similar Kaplan–Meier survival curve analysis was also performed in 178 patients without RAI therapy. The area under the curve (AUC) value was used to predict the accuracy of risk score and related RNA target, which was then further validated by the time-dependent receiver operating characteristic (tROC) curve analysis for 1, 3, and 5 years.

### Construction and Estimation of a Nomogram for PTC Patients Treated With RAI

First, the multivariate Cox regression analysis was applied to evaluate the risk scores and clinicopathological features (age, gender, neoplasm disease stage, metastasis stage, lymph node stage, tumor stage, and BRAF mutation) to determine the independent risk factor of PFS. Next, a nomogram model was constructed based on these results using the rms R package. Finally, the tROC curve analysis evaluated the predictive accuracy of the nomogram.

### Statistical Analysis

DE analysis, univariate and multivariate Cox regression, and Kaplan–Meier survival curve and tROC curve analyses were conducted using R software. The clinical features were compared between RAI-sensitive and RAI-refractory groups using a chi-square (*χ* ^2^) test in SPSS software v 24.0 using two-sided comparisons, and results with P < 0.05 were statistically significant.

## Results

### Sample Characterization

mRNA- and miRNA-seq data of 506 PTC tumor and 67 para-cancerous tissue samples, along with the respective clinical information were retrieved from TCGA database. Among 506 PTC patients, 292 patients received RAI therapy. Out of them, 254 patients showed RAI sensitivity, while 38 subjects exhibited RAI resistance. The clinical features of PTC patients with RAI therapy, including age, gender, BRAF, tumor stage, lymph node stage, metastasis stage, and neoplastic disease stage were compared by the *χ* ^2^ test between RAI-sensitive and RAI-refractory groups, which showed significant differences in tumor staging (P = 0.005) and neoplastic disease staging (P = 0.003). Additionally, patients with advanced tumor stage and neoplastic disease stage typically were presented with a higher RAI-refractory rate (**Table 1**).

**Table 1:** Characteristics of PTC patient cohorts with RAI therapy.

Characteristics	RAI-refractory group	RAI-sensitive group	P value
Total	38	254	
Gender			0.212
Female	22 (57.89%)	173 (68.11%)	
Male	16 (42.11%)	81 (31.89%)	
Age			0.201
*≤*40	11 (28.95%)	101 (39.76%)	
>40	27 (71.05%)	153 (60.24%)	
BRAF			0.101
Mutation	22 (57.89%)	111 (43.70%)	
Wild	16 (42.11%)	143 (56.30%)	
Tumor stage			**0.005**
T1+T2	12 (31.58%)	142 (55.91%)	
T3+T4	26 (68.42%)	112 (44.09%)	
Lymph node stage			0.710
N0	12 (31.58%)	88 (34.65%)	
N1+Nx	26 (68.42%)	166 (65.35%)	
Metastasis stage			0.341
M0	16 (42.11%)	128 (50.39%)	
M1+Mx	22 (57.89%)	126 (49.61%)	
Neoplastic disease stage			**0.003**
I + II	14 (36.84%)	158 (62.20%)	
III + IV	24 (63.16%)	96 (37.80%)	

Bold values indicate that the P value is less than 0.05.

### Identification of DEmRNAs and DEmiRNAs Associated With RAI Response

**Figure 2:**
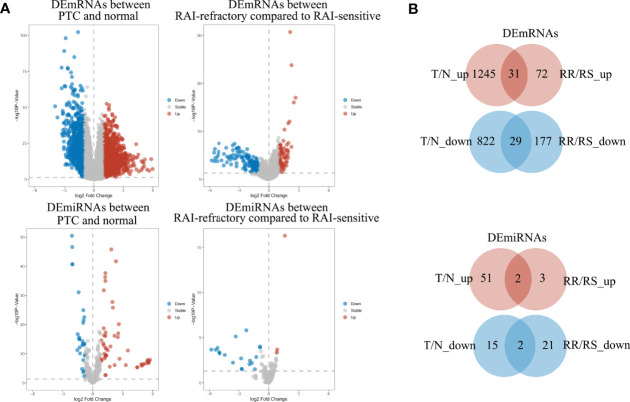
The volcano plots and Venn diagrams showed the DEmRNAs and DEmiRNAs in two comparisons. **(A)** Volcano plots of DEmRNA and DEmiRNA expressions in the PTC group compared to normal groups (T/N) and in the RAI-refractory group compared to the RAI-sensitive group (RR/RS). Red represents upregulated RNAs, and blue indicates downregulated RNAs. **(B)** Venn diagrams showed the overlap of significantly up- and downregulated DEmRNAs and DEmiRNAs in the two comparisons above.

To identify the mRNAs and miRNAs related to RAI refractoriness, DE analysis was applied to two comparisons of tumor vs. normal (T/N) and RAI-refractory vs. RAI-sensitive (RR/RS). The distributions of DEmRNAs and DEmiRNAs in these two comparisons were visualized by volcano plots (**Figure 2A**). We first identified the DEmRNAs and DEmiRNAs between PTC tumor and para-cancerous tissues, of which 2,067 mRNAs met the inclusion criteria (|log2 FC| ≥1 and P < 0.05), including 822 down- and 1,245 upregulated mRNAs, and a total of 66 miRNAs were equally eligible (|log2 FC| ≥0.8 and P < 0.05), including 15 down- and 51 upregulated miRNAs. Then, PTC samples with RAI treatment were classified into two subgroups, comprising RAI-refractory and RAI-sensitive groups based on RAI therapy results. Afterward, DEmRNA and DEmiRNA analyses were carried out between the two groups (P < 0.05 and |log FC| ≥1.0 as the mRNA thresholds; and P < 0.05 and |log FC| ≥0.8 for miRNA cutoff). Subsequently, 249 mRNAs (72 up- and 177 downregulated) and 24 miRNAs (3 up- and 21 downregulated) were screened out from RAI-refractory vs. RAI-sensitive groups. Finally, by intersecting the up- and downregulated RNAs derived from two differential analyses, we obtained 60 DEmRNAs and 4 DEmiRNAs specific to PTC RAI refractoriness, of which 31 were up- and 29 were downregulated mRNAs, while 2 were up- and 2 were downregulated miRNAs, respectively (**Figure 2B**).

### Determination of Potential Functions and Related Pathways of Target RNAs in Response to RAI

**Figure 3:**
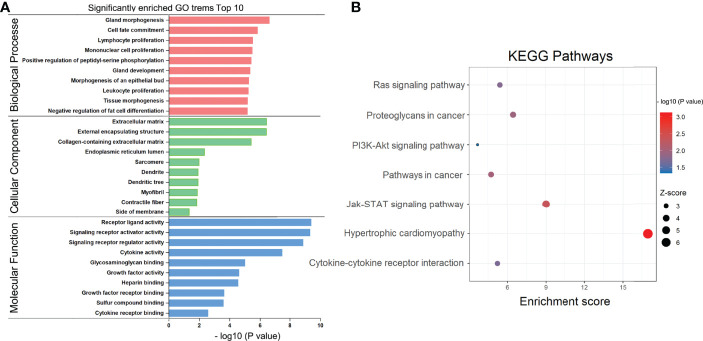
GO and KEGG pathway enrichment analysis. **(A)** Bar chart of gene ontology (GO) enriched in the biological process, cellular component, and molecular function. **(B)** Bubble diagram of KEGG pathways enrichment analysis.

To examine the putative functions and mechanistic pathways in the pathogenesis of RAI-refractory PTC, the overlapping 60 DEmRNAs were further measured through KEGG pathway and GO enrichment analyses. The Metascape tool yielded a total of 327 GO components, of which 304 were BPs, 16 were MFs, and 7 were CCs. In the BP category, most genes were involved in gland morphogenesis, cell fate commitment, lymphocyte proliferation, mononuclear cell proliferation, and positive regulation of peptidyl-serine phosphorylation. In the MF category, most genes were associated with receptor ligand activity, signaling receptor activator/suppressor activity, glycosaminoglycan binding, and cytokine activity. In the CC category, a large proportion of genes were linked to the extracellular matrix, endoplasmic reticulum lumen, external encapsulating structure, sarcomere, and collagen-containing extracellular matrix. The top 10 significantly enriched GO classification terms are displayed in **Figure 3A**. Furthermore, KEGG pathway analysis identified 7 pathways with significant enrichment (**Figure 3B**), such as hypertrophic cardiomyopathy, Jak-STAT signaling pathway, cytokine–cytokine receptor interaction, PI3K-Akt signaling pathway, pathways in cancer, proteoglycans in cancer, and Ras signaling pathway. The most significantly enriched was hypertrophic cardiomyopathy, involving 3 downregulated genes (*DES*, *IL6*, and *MYL2*). These results present critical clues to the mechanism of RAI-refractory PTC pathogenesis.

### Selection of Prognosis-Related RNAs Associated With RAI Response

**Figure 4:**
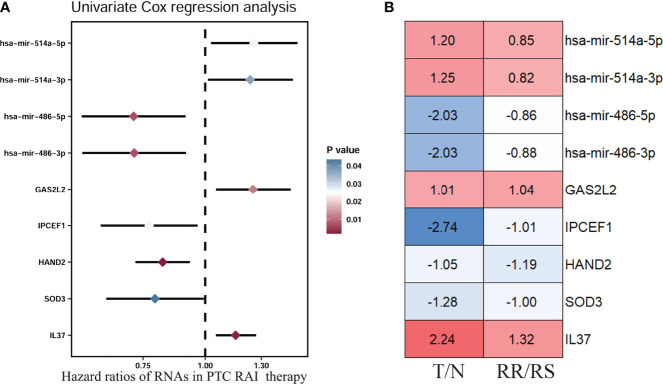
Prognostic value of single-candidate RNA. **(A)** The forest map results of the univariate Cox regression analysis of the nine screened variables in PTC patients who received RAI therapy. **(B)** Module diagram showing the log2 fold change (FC) of the nine RNAs between tumor vs. normal (T/N) groups and RAI-refractory vs. RAI-sensitive (RR/RS) groups.

**Figure 5:**
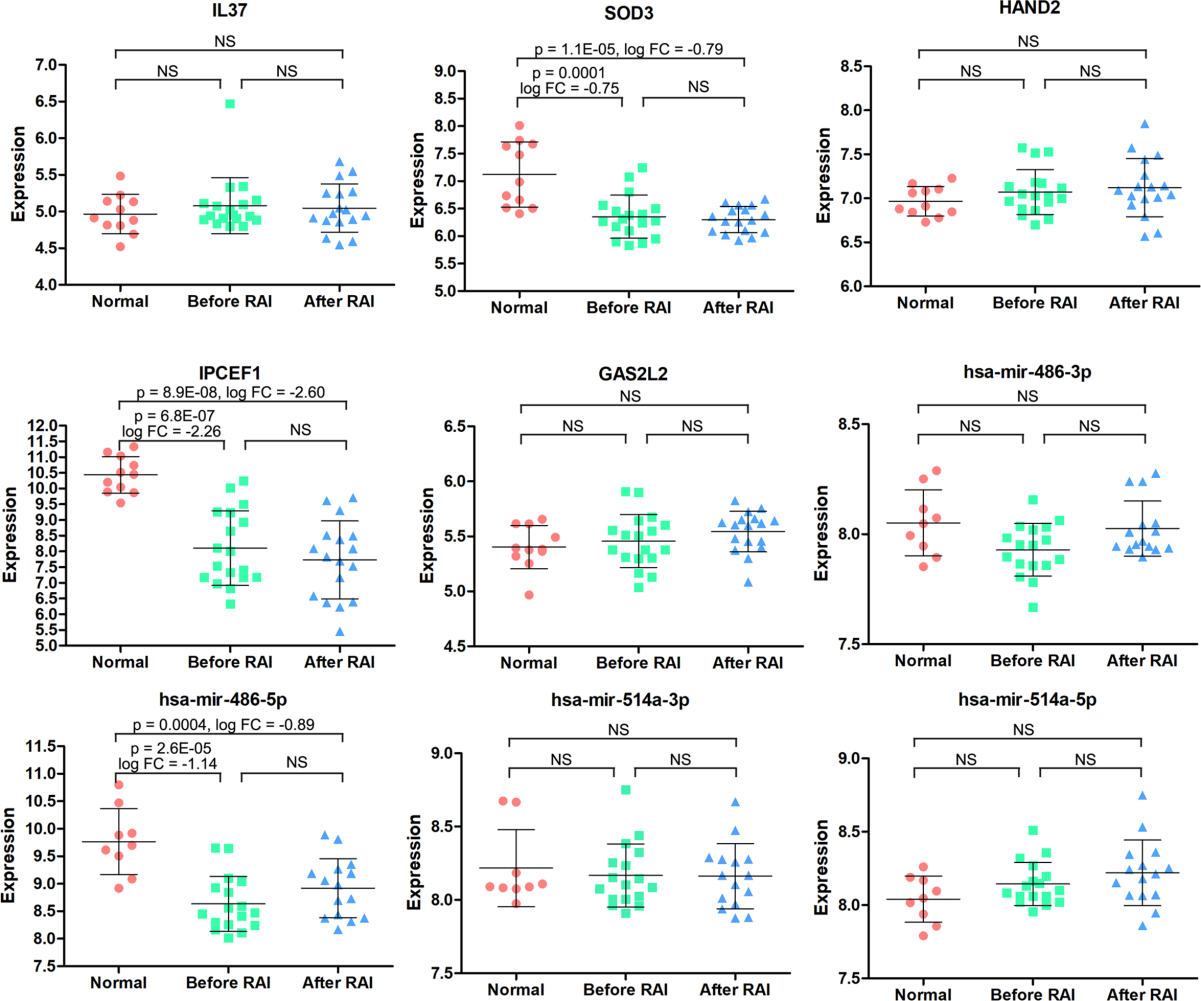
The expression patterns of the 9 candidate RNAs in normal, PTC before RAI, and PTC after RAI groups were validated in the GSE151181. NS, no significance.

So far, 60 DEmRNAs and 4 DEmiRNAs related to RAI refractoriness had been screened out. To further explore the prognostic value of single-candidate RNA, the univariate Cox regression model was applied to the PTC RAI therapy cohort (n = 289) (**Table 2**), which revealed 5 DEmRNAs (IL37, SOD3, HAND2, IPCEF1, and GAS2L2) and 4 DEmiRNAs (has-mir-514a-5p, has-mir-514a-3p, has-mir-486-5p, and has-mir-486-3p) having significant relevance with the PFS of PTC patients treated with RAI (**Figure 4A**), and the log2 FC of these RNAs in the comparisons of T/N and RR/RS is shown in **Figure 4B**. Afterward, the GEO database was searched to explore datasets about RAI-refractory PTC. Consequently, GSE151181 was the only microarray dataset associated with the above conditions. To obtain the key RNAs effective for the RAI therapy, the 9 abovementioned candidate RNAs were further validated in GSE151181. Our results illustrated that there were positive differences for IPCEF1 and hsa-mir-486-5p across the control, PTC before RAI, and PTC after RAI groups, with P < 0.05 and |log2 FC| ≥1 as the mRNA threshold, and P <0.05 and |log2 FC| ≥0.8 as the miRNA threshold (**Figure 5**). IPCEF1 and hsa-mir-486-5p were co-depleted in RAI-refractory PTC tissues.

**Table 2:** The univariate COX analysis of 64 signatures in the RAI therapy cohort.

Symbol	HR	z	P value
C1QTNF12	1.165	1.358	0.175
TAS1R1	1.173	1.807	0.071
PLA2G2E	1.081	1.431	0.153
CYP4B1	1.137	1.263	0.207
VTCN1	1.080	1.466	0.143
SPRR1B	1.045	0.897	0.370
FAM163A	0.957	-0.561	0.574
RGS8	0.877	-1.828	0.068
B3GALT2	0.934	-0.780	0.435
CAMK1G	1.085	0.851	0.395
WNT3A	1.090	1.288	0.198
TRIM54	1.092	0.865	0.387
SLC5A7	0.885	-1.954	0.051
IL37	1.155	2.979	**0.003**
GALNT5	1.076	0.967	0.334
DES	0.898	-1.462	0.144
CADPS	1.113	1.483	0.138
ADIPOQ	0.995	-0.087	0.931
APOD	0.855	-1.818	0.069
SOD3	0.793	-2.019	**0.043**
NWD2	0.980	-0.296	0.767
ADH1B	0.879	-1.960	0.050
NPY5R	0.901	-1.825	0.068
HAND2	0.821	-3.060	**0.002**
FGF10	1.019	0.350	0.726
KCNIP1	0.956	-0.743	0.457
RBM24	0.902	-1.114	0.265
CLPSL2	0.980	-0.262	0.793
IPCEF1	0.771	-2.267	**0.023**
PNLDC1	1.030	0.383	0.702
TBXT	1.094	1.684	0.092
SOSTDC1	0.929	-1.113	0.266
IL6	0.911	-1.248	0.212
DLX6	0.854	-1.306	0.192
COL26A1	1.198	1.786	0.074
AOC1	1.138	1.832	0.067
SHH	0.903	-1.511	0.131
CSAG1	1.071	0.878	0.380
CSMD1	1.095	1.293	0.196
GDF6	1.049	0.787	0.431
CCN4	1.062	0.807	0.420
IFNE	0.953	-0.721	0.471
CA9	1.033	0.439	0.661
AMBP	0.972	-0.391	0.696
OBP2B	1.038	0.626	0.531
RAG2	0.916	-1.316	0.188
FAM180B	0.906	-1.678	0.093
TCN1	1.050	0.791	0.429
MMP10	1.046	0.766	0.444
SPAG6	1.069	1.144	0.253
GDF10	0.916	-1.292	0.196
NTS	0.939	-1.283	0.199
MYL2	0.916	-0.783	0.433
NOS1	1.006	0.066	0.947
PIWIL1	1.044	0.639	0.523
SERTM1	0.955	-0.761	0.447
SERPINA5	0.984	-0.272	0.786
GAS2L2	1.251	2.520	**0.012**
CSF3	0.918	-1.454	0.146
CD300LG	0.907	-1.344	0.179
hsa-mir-486-3p	0.720	-2.685	**0.007**
hsa-mir-486-5p	0.718	-2.696	**0.007**
hsa-mir-514a-3p	1.235	2.085	**0.037**
hsa-mir-514a-5p	1.256	2.216	**0.027**

Bold values indicate that the P value is less than 0.05.

**Figure 6:**
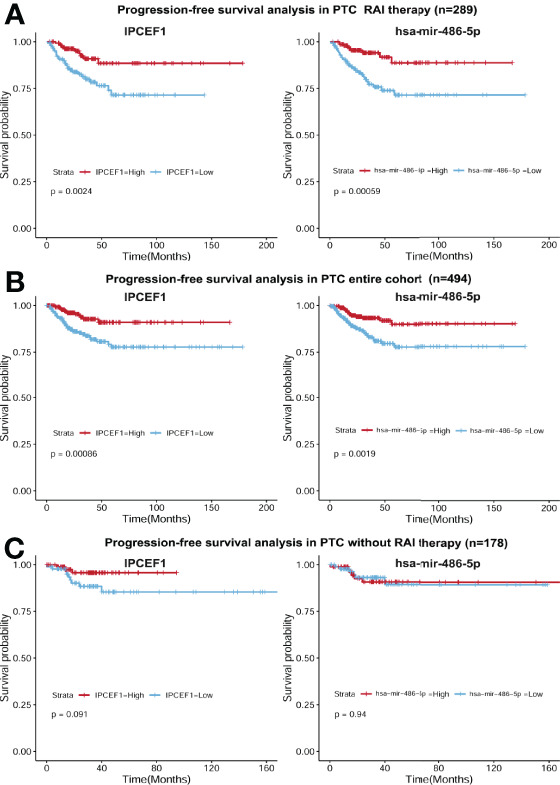
Survival curves of PFS in the PTC RAI therapy cohort **(A)**, in the all-PTC group **(B)** and in PTC without RAI therapy **(C)** with high and low expressions of IPCEF1 and hsa-mir-486-5p.

Next, to determine whether these two candidate RNAs were associated with PTC prognosis, we employed Kaplan–Meier survival curve analysis with the LR test to estimate PFS in the PTC RAI therapy cohort (n = 289), without RAI therapy cohort (n = 178), and the entire PTC cohort (n = 494). Our study identified that IPCEF1 and hsa-mir-486-5p were related to prognosis not only in PTC with RAI therapy but also in all PTC groups (**Figures 6A, B** **)**. However, in PTC without RAI therapy, neither of them was related to the PFS (**Figure 6C**). This result demonstrated that the higher expression of IPCEF1 and hsa-mir-486-5p was apparently interrelated with the better PFS of PTC patients with RAI therapy.

### Construction of a Prognostic Risk Model for PTC Patients Treated With RAI

**Figure 7:**
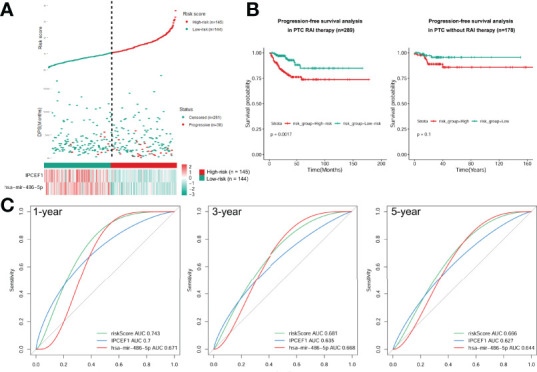
Construction of a prognostic risk model. **(A)** Risk score based on the 2 signatures, survival status, and heatmap for the expressions of them for each RAI therapy PTC patient. **(B)** PFS difference between high-risk and low-risk groups of PTC with RAI therapy or without RAI therapy using the Kaplan–Meier curve. **(C)** Time-dependent ROC curves of the risk score were adopted to estimate the predictability of PFS at 1, 3, and 5 years.

Next, we calculated the risk score for prognosis of RAI therapy patients with IPCEF1 and hsa-mir-486-5p as risk score = (-0.2288 × IPCEF1 expression level) +(-0.2969 × hsa-mir-486-5p expression level). Afterward, the RAI therapy patients were subdivided into the low-risk (n = 144) and high-risk (n = 145) groups, as per their median risk score (**Figure 7A**). Moreover, the progressive disease rates of RAI therapy patients were up ticked with the prognostic risk score and the heatmap analysis revealed that IPCEF1 and hsa-mir-486-5p had reduced expressions in the high-risk group (**Figure 7A**). Evidently, the Kaplan–Meier survival curve showed that the high-risk group had worse PFS compared with the low-risk group in the RAI therapy cohort (P = 0.0017). However, there was no significant difference in the cohort without RAI therapy (P = 0.1) (**Figure 7B**). The predictive accuracy of the risk score was further assessed by the tROC curves in the RAI therapy cohort (n = 289), with 1-, 3-, and 5-year AUCs of 0.743, 0.681, and 0.666, respectively, which indicated more precise AUC values compared with single RNA (IPCEF1: 0.7, 0.635, and 0.627; hsa-mir-486-5p: 0.671, 0.668, and 0.644) (**Figure 7C**). These results suggested that the multi-RNA model had more efficient prediction ability in PTC patients with RAI therapy.

### Establishment and Estimation of a PFS-Predicting Nomogram for PTC Patients With RAI Treatment

**Figure 8:**
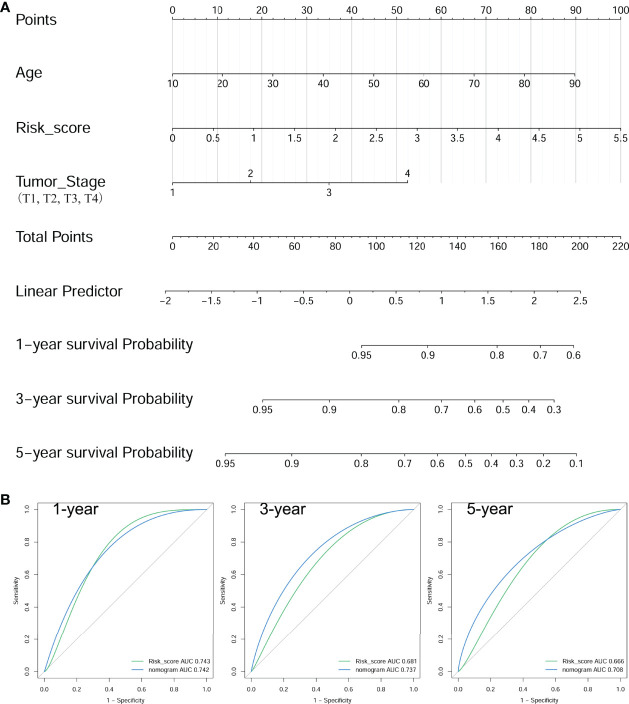
Construction of a nomogram for PFS in PTC patients with RAI therapy. **(A)** The baseline nomogram from age, risk score, and tumor stage. **(B)** tROC curves of the nomogram for predicting the PFS rates at 1, 3, and 5 years.

The multivariate Cox regression model analyzed clinicopathological features (including age, gender, neoplastic disease stage, metastasis stage, lymph node stage, tumor stage, and BRAF mutation) and risk score for each group (**Table 3**). Results presented as hazard ratio (95% CI)-risk score (1.494 [1.069–2.088]), age (1.025 [1.004–1.047]), and tumor stage (1.471 [0.991–2.182]) were independently associated with PFS in PTC cases with RAI therapy. Furthermore, the nomogram model was established to quantitatively predict PFS based on the abovementioned factors. One point was allocated for each factor, then the total point was obtained by summing up all factors to estimate PFS rates at 1, 3, and 5 years (**Figure 8A**). Comparisons of tROC curves for nomogram and risk scores showed that the AUC values of the 3- and 5-year PFS of the nomogram were higher than those of risk scores (0.737 vs. 0.681, 0.708 vs. 0.666, respectively), and there was no difference with respect to 1-year PFS (0.742 vs. 0.743), suggesting a better predictive capacity of the prognostic nomogram after adding the risk score, age at diagnosis, and tumor stage to predict PFS (**Figure 8B**). Results indicated that the nomogram had better 3- and/or 5-year PFS predictions than risk scores in PTC patients with RAI therapy.

**Table 3:** Multivariate analysis of the progression-free survival in RAI therapy cohort.

Variable	HR	95%CI	P value
Risk score	1.494	1.069–2.088	**0.019**
Age	1.025	1.004–1.047	**0.021**
Gender	0.887	0.454–1.732	0.184
TNM stage	1.175	0.777–1.777	0.113
Metastasis stage	1.069	0.766–1.490	0.176
Lymph node stage	1.026	0.598–1.758	0.235
Tumor stage	1.471	0.991–2.182	**0.055**
BRAF mutation	1.308	0.668–2.563	0.110

Bold values indicate that the P value is less than 0.05.

## Discussion

Although patients with PTC may be clinically indolent and have a good prognosis after RAI treatment (6, 29, 30), however, the dedifferentiation of PTC tumor cells can take place during disease progression becoming RAI resistance, which is negatively associated with the treatment outcome (31, 32). Although remarkable advancements have been made in the targeted therapy against RAI-refractory PTC, the ultimate effects are still unsatisfactory (4). Therefore, construction of an early prognostic model for RAI-refractory PTC patients is critical to allowing efficient treatments and preventing tumor progression. In this study, we particularly concentrated on PTC patients with RAI treatment, which was innovative and disparate from previous studies. We successfully constructed a nomogram model to accurately predict the RAI refractoriness in PTC patients.

According to KEGG enrichment analysis, we found that a total of 7 pathways were significantly enriched. The hypertrophic cardiomyopathy pathway, Jak-STAT signaling pathway, and Ras signaling pathway were identified as the three most significant pathways in RAI-refractory PTC cases. The hypertrophic cardiomyopathy pathway was shown to be significantly connected with breast and colorectal cancer (33, 34), but the biological function was unknown. After total thyroidectomy, it is necessary for PTC patients to take levothyroxine as a replacement therapy. Some of them, especially those with a high risk of recurrence, are in subclinical hyperthyroidism. Mastroianno et al. found that subclinical hyperthyroidism could cause hypertrophic cardiomyopathy (35). Janus kinases (JaK) are a family of tyrosine kinases (TKs), including JAK1, JAK2, JAK3, and TYK2, and all of their receptors actively participate in the pathogenesis of various human cancers (36). Previous evidence presented the role of the Jak-STAT signaling pathway in cancer cell proliferation, differentiation, death, and survival (36). Bi et al. had demonstrated that JAK1 in PTC tissues was prominently upregulated than that in adjacent normal tissues, and its expression level was associated with tumor differentiation, lymph node metastasis, invasion degree, and TNM stage, and upregulated JAK1 influenced the progression of PTC (37). Ras protein is a classical molecular switch, turning between off and on states during signal transduction. RAS gene mutation abrogates its switching capacity leaving it in a constitutively active state, promoting diseased conditions (38, 39). It has been shown that the Ras signaling pathway plays significant roles in cellular differentiation, proliferation, apoptosis, and carcinogenesis (38–40). In addition, Ras can also activate the MAPK and PI3K/AKT pathways, leading to the progression of thyroid cancer (40). Interestingly, when analyzing the DEmRNAs of the three enrichment pathways mentioned above, we found that IL6 was downregulated in all of them. IL-6 is a multifunctional cytokine participating in cell growth and differentiation, inflammatory reaction, and tumor growth (41, 42). It is considered that IL-6 mainly plays a role by activating the JAK/STAT signaling pathway through GP130 after binding to its receptor (41). Currently, the role of IL-6 in thyroid cancer remains controversial. Research suggested that a higher IL-6 mRNA expression was observed in the PTC tissues than in the adjacent normal tissues (42), while Basolo et al. found that IL-6 was downregulated in undifferentiated TC tissues compared to well-differentiated tissues (43). Thus, IL-6 might be a potential marker in the mechanism of RAI-refractory PTC. However, it plays a role through the above three signaling pathways which require further experiments.

Through the univariate Cox regression analysis, we found that 5 mRNAs (IL37, SOD3, HAND2, IPCEF1, and GAS2L2) and 4 miRNAs (has-mir-514a-5p, has-mir-514a-3p, has-mir-486-5p, and has-mir-486-3p) were correlated with the prognostic rate in PTC patients with RAI therapy. Previous studies have observed that IL37 and SOD3 expressions were connected with poor prognosis in PTC (24, 44). Regrettably, IL37 and SOD3 genes did not enter our final prognostic model. Consequently, following GSE151181 microarray analysis, both mRNA IPCEF1 and miRNA hsa-mir-486-5p had been validated as biomarkers which could effectively predict the PFS of patients from both groups.

IPCEF1 is translocated to the plasma membrane in response to growth factor (GF) signaling to enhance the exchange activity of cytohesin-2 (45, 46). IPCEF1 is homologous to the CNK3 C-terminal domain, which is involved in signal transduction downstream of Ras (47). The MAPK signaling pathway promotes dedifferentiation in PTC with constitutive activation of Ras mutation (48). Additionally, studies have shown that IPCEF1 induces tumor metastasis by activating the Arf6 pathway (49–51). Espinal-Enríquez and coworkers discovered that IPCEF1 was underexpressed in thyroid cancer (50). Schulten et al. observed a lower expression of IPCEF1 in PTC, especially follicular subtype, compared to normal thyroid samples (52). In our study, the IPCEF1 expression is significantly lower in PTC than in para-cancerous tissues, and a lower expression of IPCEF1 is relevant to a worse prognosis of PTC patients with RAI therapy.

hsa-mir-486 is located within the last intron of the ankyrin-1 gene on the chromosome 8p11’s short arm. We demonstrated that downregulation of hsa-mir-486-5p was significantly associated with PTC progression. Several studies have demonstrated the role of hsa-mir-486-5p in numerous human cancers (53–57). For instance, mir-486-5p was markedly downregulated in non-small cell lung cancer (NSCLS), gastric cancer, hepatocellular carcinoma, and colorectal and pancreatic cancer (53, 54, 58–60). Inversely, mir-486-5p was upregulated in chronic myelocytic leukemia (CML) and cervical cancer (56, 61). Hence, mir-486-5p might be differentially regulated depending on the type of cancer. Ma et al. have shown that mir-486-5p undergoes downregulation in PTC tissues and is negatively correlated with Fibrillin-1 (FBN1) mRNA levels *in vivo* and *in vitro*. A decreased expression of mir-486-5p led to tumor proliferation, growth, and progression by targeting FBN1 (62). Here, we showed the decreased expression of hsa-mir-486-5p in PTC and demonstrated a prognostic value for PTC patients with RAI therapy. Thus, we extended the potential role of IPCEF1 and hsa-mir-486-5p in RAI-refractory PTC cases.

However, our study still has certain limitations: 1) because PTC patients have a longer survival period and fewer patients have observed death outcomes, we could only perform PFS analysis; 2) the nomogram model only played a predictive role, and a validation cohort was not available; and 3) information on RAI dose is not available and thus cannot rule out differences in RAI dose influence response.

In summary, we established a risk model according to two prognostic RNA signatures (IPCEF1 and hsa-mir-486-5p) from publicly available datasets and then constructed a nomogram including clinical parameters (tumor stage and age at diagnosis) to predict 1-, 3-, and 5-year PFS in PTC patients with RAI therapy, which might be applied as a potential prognostic signature in strategizing clinical practice for personalized treatment.

## Data Availability Statement

The datasets presented in this study can be found in online repositories. The names of the repository/repositories and accession number(s) can be found in the article/supplementary material.

## Author Contributions

WT and ZJ conceived the study idea. QL, YH, and DX collected the data to be analyzed. QLa, XJ, QLi, and YX performed the data analysis and produced the results. LL, YS, and WT wrote and revised the manuscript. All authors contributed to the article and approved the submitted version.

## Funding

This work was supported by the Scientific Research Fund of Chengdu Medical College (CYZ18-12), Project of Science & Technology Department of Sichuan Province (2020JDRC0134), Innovation and Entrepreneurship Training Program for College Students, Chengdu Medical College (S201913705101), Scientific Research Project of Medicine Department of Sichuan Province (S18001), Health Commission of Sichuan Province (20PJ227), Scientific Research Project of Medicine Department of Chengdu City (2021051 and 2021135), and Special Project of “Technological innovation” Project of CNNC Medical Industry Co. Ltd. (ZHYLYB2021003).

## Conflict of Interest

The authors declare that the research was conducted in the absence of any commercial or financial relationships that could be construed as a potential conflict of interest.

## Publisher’s Note

All claims expressed in this article are solely those of the authors and do not necessarily represent those of their affiliated organizations, or those of the publisher, the editors and the reviewers. Any product that may be evaluated in this article, or claim that may be made by its manufacturer, is not guaranteed or endorsed by the publisher.
